# Synchronous triple primary gastrointestinal malignant tumors treated with laparoscopic surgery: A case report

**DOI:** 10.1515/med-2023-0742

**Published:** 2023-07-03

**Authors:** Wei Jiang, Genshan Zhang, Haijie Li, Xiangshang Xu, Lingwei Jia, Xuelai Luo, Zhixin Cao

**Affiliations:** Department of Gastrointestinal Surgery, Tongji Hospital of Tongji Medical College, Huazhong University of Science and Technology, 430000, Wuhan, China; Department of Gastrointestinal Surgery, Tongji Hospital of Tongji Medical College, Huazhong University of Science and Technology, No. 1095 Jiefang Avenue, 430000, Wuhan, China

**Keywords:** case report, multiple primary malignant tumors, gastric cancer, colorectal cancer, laparoscopic surgery

## Abstract

Synchronous gastrointestinal multiple primary tumors including gastric, colonic, and rectal cancers are rare. Moreover, it was a challenge to find an appropriate procedure without negatively impacting the overall outcome. We described the case of a 63-year-old woman who presented with a 4 month history of upper abdominal pain, acid regurgitation, and anemia. Gastroscopy with biopsy suggested early cancer of gastric antrum. Abdominal contrast-enhanced computerized tomography and colonoscopy revealed ascending colon and rectum tumors. She had no family history of malignancy. Endoscopic submucosal dissection was performed for gastric cancer, and the pathological result presented that it was poorly differentiated and invaded into deep submucosa. The laparoscopy-assisted radical surgery combined with distal gastrectomy, right hemicolectomy, and anterior resection of rectum was performed for these three tumors via eight ports and a 7 cm midline upper-abdominal incision. No other perioperative complications were encountered except postoperative ileus. The patient was discharged on the 12th postoperative day. The pathological results revealed gastric cancer (T1N0M0), right colonic cancer (T3N1M0), and rectal cancer (T2N0M0), indicating complete surgical resection. We reported that our laparoscopic approach for synchronous triple primary gastrointestinal malignant tumors was feasible and minimally invasive.

## 1 Introduction

Multiple primary tumors (MPT) are defined as more than one tumor diagnosed in the same patient. The incidence of multiple primaries in cancer patients is reported in the range of 2–17% [1]. Depending on different definitions, synchronous MPT is defined as tumors diagnosed within 2 or 6 months of the initial diagnosis of a primary tumor [2,3]. Synchronous triple primary malignant tumors are a very rare finding [4] with uncertain incidence. Extensive surgery might lead to worse postoperative outcomes. The challenge was to find an appropriate procedure without a negative impact on the overall outcome when synchronous malignancies were diagnosed [1,5]. Laparoscopic surgery had been widely used for gastrointestinal cancer with many advantages over traditional open surgery [6]. However, there were few reports about laparoscopic procedures used in MPT [7]. And in terms of triple primary malignancies, there were no such reports yet. Here we reported a case of laparoscopic resection of triple gastrointestinal tumors via our procedure successfully.

## 2 Case presentation

A 63-year-old woman visited the Department of Gastroenterology because of upper abdominal pain with acid regurgitation and anemia for 4 months. Her past medical history included a hysterectomy for benign disease and lumbar disc prolapse. Her body mass index was 27.8 kg/m^2^. A gastroscopy was performed and suggested an early-stage gastric cancer lesion located at the antrum with shallow ulcer formation, about 1.5 cm in diameter. The pathology confirmed gastric adenocarcinoma. Abdominal contrast-enhanced computerized tomography (CT) showed a space-occupying lesion of the ascending colon and thickening of the rectal wall with no metastatic lesions. Furthermore, colonoscopy with biopsy revealed ascending colonic cancer with stenosis as well as rectal cancer of 2.5 cm in diameter located 12 cm proximal to the anal verge. Laboratory tests showed elevated serum carbohydrate antigen (CA-199) (30.39 U/mL). Additionally, preoperative chest CT found no lung metastasis.

Given the possible diagnosis of early-stage gastric cancer, endoscopic submucosal dissection was performed. However, the pathological result presented that it was poorly differentiated and invaded into deep submucosa. Therefore, it was required to manage three tumors with minimal adverse effects; and laparoscopic surgery was considered.

**Figure 1:**
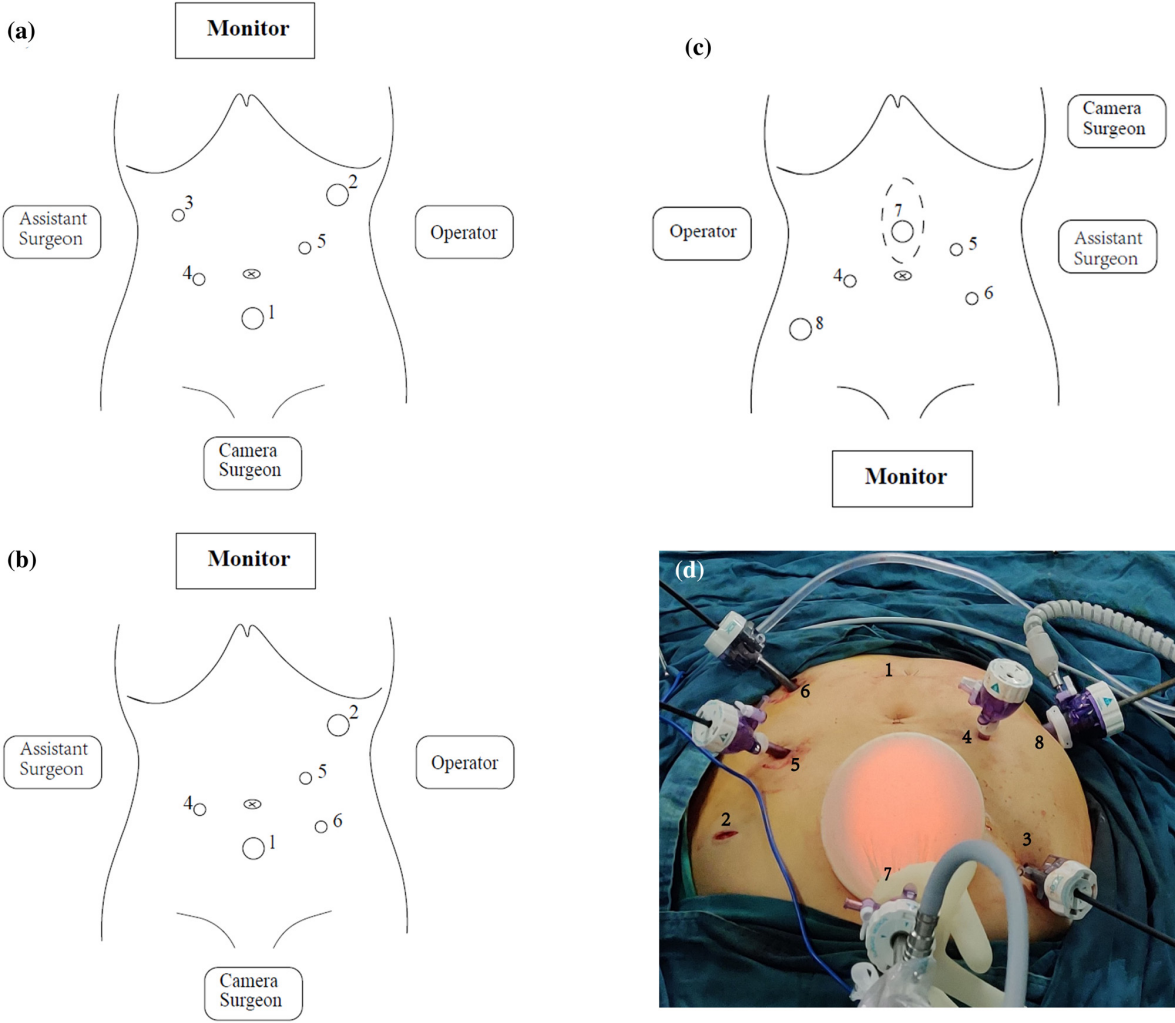
The steps of laparoscopic surgery for synchronous multiple gastrointestinal cancer. The patient was placed in the lithotomy position. The surgeons’ position and the trocar ports were introduced as shown. (a) The positions for distal gastrectomy; (b) the positions for right hemicolectomy; and (c) the positions for anterior resection of rectum. (d) The position of the incision and ports.

Laparoscopic surgery was performed under general anesthesia, and the patient was placed in the lithotomy position. Pneumoperitoneum was established through a Veress needle which was inserted just above the umbilicus. The intra-abdominal pressure was maintained at the level of 12 mmHg. Then a 10 mm port (Port 1) was introduced at the point about 5 cm under the umbilicus for a 30 degree laparoscopic camera. After abdominal exploration, the patient was turned into lithotomy-anti-Trendelenburg position. The first 12 mm port (Port 2) and the first 5 mm port (Port 3) were introduced below the costal margin at the left and right anterior axillary lines, respectively. The position of Port 3 was a little closer to the foot side than Port 2. Then, another 5 mm port (Port 4) was introduced above umbilicus level at left midclavicular lines. Moreover, one more 5 mm port (Port 5) was introduced under the umbilicus level at the left midclavicular line. These five ports were used to perform the right-side approach laparoscopic distal gastrectomy with D2 lymphadenectomy (Figure 1a). After the separation and dissection of the distal gastrectomy, another 5 mm port (Port 6) was introduced under the umbilicus level at the right midclavicular line. Ports 1, 3, 4, 5, and 6 were used to perform the separation and dissection of right hemicolectomy with D3 lymphadenectomy (Figure 1b). Then a 7 cm midline upper abdominal incision, just above the umbilicus, was made for anastomosis of distal gastrectomy and right hemicolectomy. A disposable wound retractor was used for incision protection. Billroth II reconstruction with 29 mm circular stapler was chosen as the type of gastrointestinal reconstruction, while end-to-side anastomosis with 29 mm circular stapler was the reconstruction type of right hemicolectomy.

**Figure 2:**
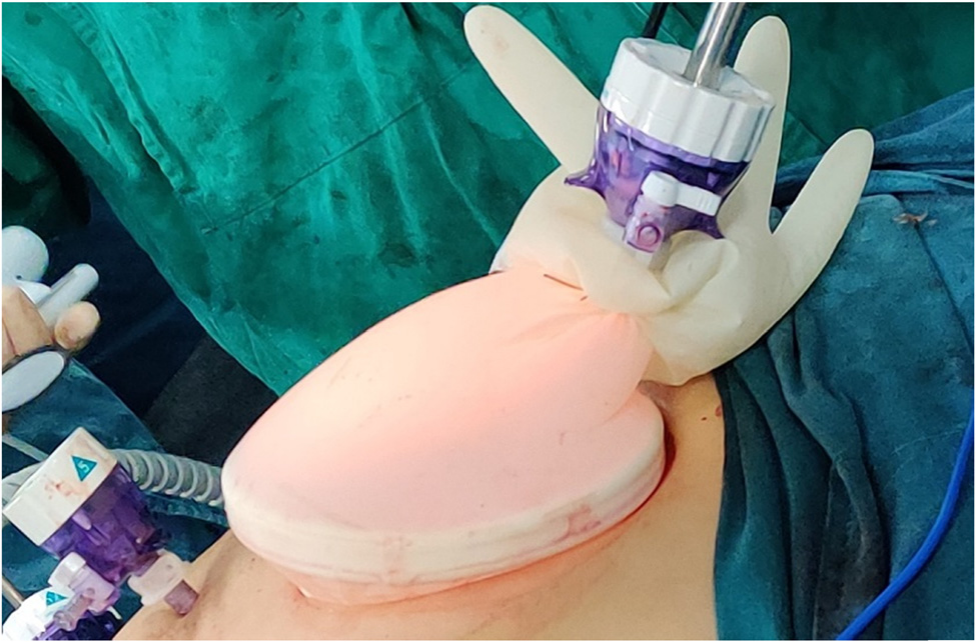
The unit for the pneumoperitoneum reestablishing and the laparoscopic camera inserting. The sac wrist of a glove was used to snare the disposable wound retractor. The thumb of the glove was cut off, and a 10 mm port was inserted into the thumb stump. Then, the thumb stump was ligatured by a 1-0 suture.

**Figure 3:**
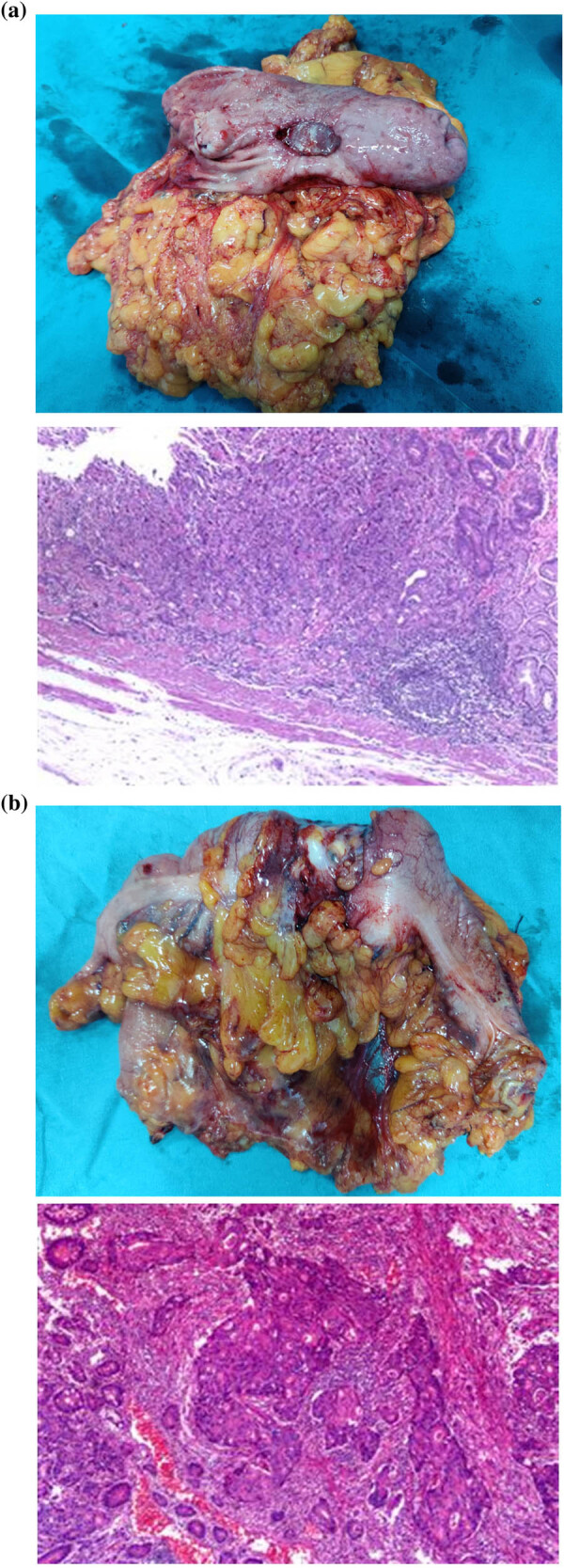

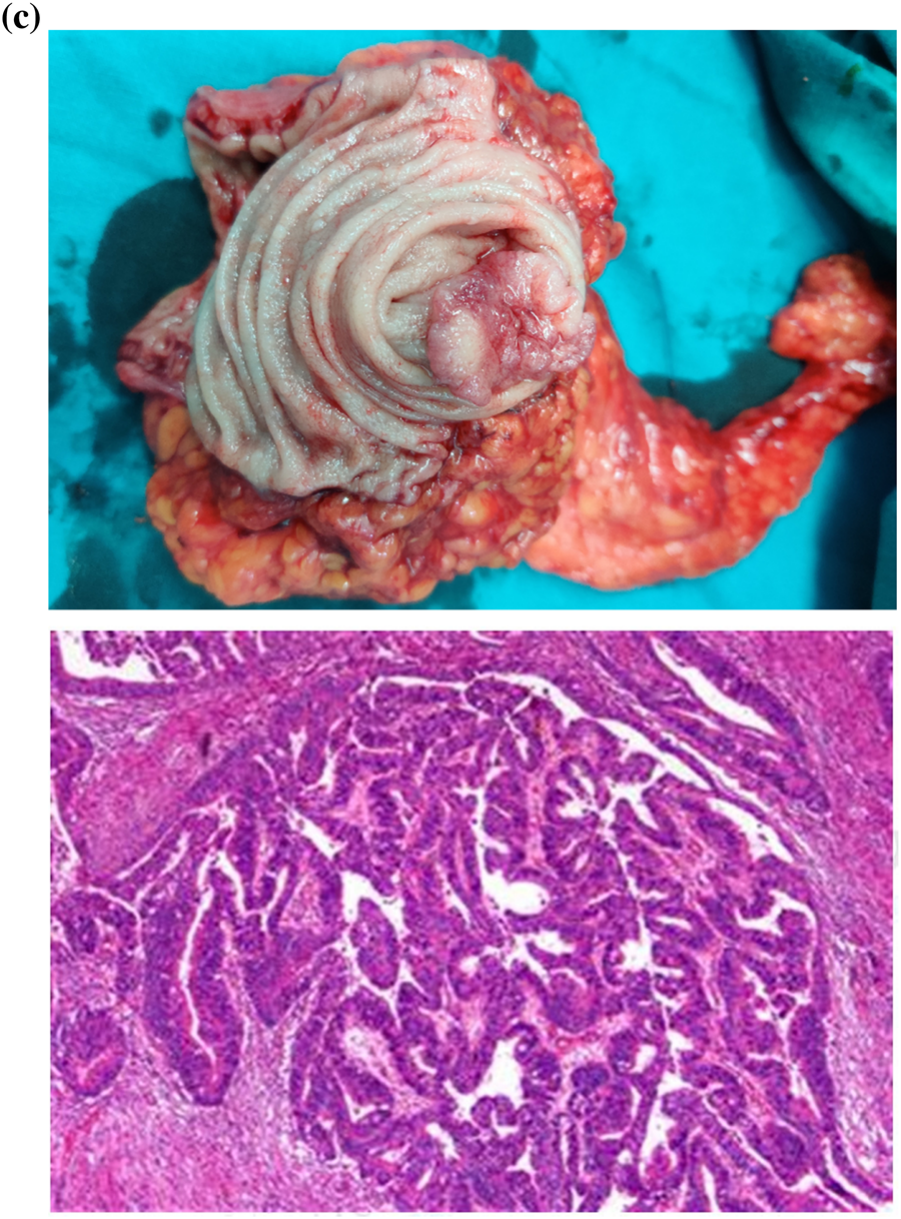
Gross specimens and microscopic morphologic pictures of three tumors. (a) distal gastrectomy; (b) right hemicolectomy; and (c) anterior resection of rectum.

Then, the sac wrist of a glove was used to snare the disposable wound retractor. The glove’s thumb was cut off, and a 10 mm port (Port 7) was inserted into the thumb stump (Figure 2). After that, the thumb stump was ligatured by a 1-0 suture. This unit, just like the single port laparoscopy device, was used for the laparoscopic camera inserting and the pneumoperitoneum reestablishing. The patient was turned into lithotomy-trendelenburg position. Another 12 mm port (Port 8) was introduced at McBurney’s point. Thus, Ports 4, 5, 6, 7, and 8 were used to perform the separation and dissection of the anterior resection of rectum (tumor-specific mesorectal excision) (Figure 1c). After stopping the inflation and removing the glove, the incision could be used to perform the anastomosis of the rectum and colon with a 29 mm circular stapler. The gross specimens and microscopic morphologic pictures of three tumors are shown in Figure 3.

The duration of surgery was 330 min with a blood loss of 200 mL. The only postoperative complication was postoperative ileus. The patient’s first flatus and stool time were on day 4 and 6 after surgery, respectively. Because of distension, enteral feeding remained a low amount before day 7 and increased to about 80% of target needs on day 10 after surgery. Supplement parental nutrition support was started on day 5 and stopped on day 11 after surgery. The patient was discharged and returned home on day 12 with no other complications.

The pathological results revealed stage T1N0M0 gastric adenocarcinoma, stage T3N1M0 adenocarcinoma of the ascending colon, and stage T2N0M0 adenocarcinoma of rectum. And deficient mismatch repair (dMMR) was not found on immunohistochemistry (IHC) testing, which indicated microsatellite stability. The patient received capecitabine and oxaliplatin as postoperative adjuvant chemotherapy. By the time of submission of this manuscript, 1 year after surgery, follow-up CT showed no recurrence, and the patient was capable of general housework.


**Ethics approval and consent to participate:** This study was approved by the Ethics Committee of Tongji Hospital. Consent for this presentation is obtained from the patient in writing.
**Consent for publication:** The patient provided written consent for publication of his case details and medical images.

## 3 Discussion and conclusion

In general, MPT was considered rare and described in case reports [4,8]. The improvement in screening and diagnostic procedures had led to the increasing frequency of MPT [1,9]. Synchronous gastrointestinal MPT was relatively rare. The incidence varied between 1.1 and 3.5% in the population with primary gastrointestinal cancer [7,10,11]. In this report, we presented a patient with synchronous gastric, colonic, and rectal cancer, which was even rare.

For patients with resectable gastric, colonic cancer, as well as stage I and II rectal cancer, the surgical procedure was the first recommendation in clinical guidelines [12,13,14,15,16]. However, a wide incision would be needed if traditional open surgery was performed. And it might lead to a poor prognosis because of severe trauma and postoperative complications. In this report, the patient was overweight, which increased the difficulty of the surgery and the possibility of poor outcomes [17,18]. Now, laparoscopic surgery had been widely used for gastrointestinal cancer. The benefits of minimally invasive surgery in gastrointestinal surgery had been well established. And in many experienced medical centers, similar oncology and safety outcomes are achieved compared to traditional open surgery [19,20,21,22]. The observational study [5] concluded that the most likely reason for unfavorable outcomes was extensive surgery, in which the synchronous colorectal tumors were less often treated by laparoscopy. Therefore, we chose laparoscopic surgery, which was more likely tolerated by the patient.

For three tumors to resect, the steps of the whole surgery should be carefully planned. The gastrectomy and colectomy were relatively clean operations. And during these two operations, the monitor was positioned on the head side of the patient. Additionally, one single incision could be used for reconstruction for both the operations. Therefore, these two operations were performed first. In this case, the unit combined the wound retractor, glove, and the port, which was used for the laparoscopic camera and the pneumoperitoneum reestablishing in proctectomy. This simple unit could be applied in many other conditions in our center, like laparoscopic examination before the abdomen closes. In addition, the reconstruction of rectectomy was more accessible with a circular stapler. The upper abdomen incision could be used to excise the tumor and insert the anvil shaft when about 5 cm more proximal descending colon was dissociated. Hence, rectectomy was performed as the second step. For the reason of longer operative time, we did not choose the total laparoscopic approach. The laparoscopic-assisted approach for resection of three tumors would reduce operative time, leading to less complication and better prognosis [23]. Eventually, with some compromise on the placement of trocar ports and the position of incision, this complex surgery was performed successfully.

In this case, the patient had postoperative ileus as the only complication after the surgery. It might be associated with extensive surgical trauma and postoperative inflammation [24]. The postoperative hospital stay was similar to the time reported in previous studies about gastrointestinal MPT, in which the patients were diagnosed with gastrointestinal synchronous double MPT [7,25]. And the patient received postoperative adjuvant chemotherapy successfully as planned, which also indicated the safety of this surgery.

Lynch syndrome was the common cause of multiple primaries in patients with colon cancer [1,26]. However, it was excluded for the reason that dMMR was not found on IHC testing in this case. It was a limitation that the genetic testing was not performed.

We reported a single case of gastrointestinal triple primary tumors treated by laparoscopic surgery. As long as the surgical procedure details are properly planned and the indications are carefully selected, laparoscopic surgery is safe and feasible as an MPT treatment even in synchronous triple primary gastrointestinal malignant tumors.

## Abbreviations

MPT: multiple primary tumors
CT: computerized tomography
dMMR: deficient mismatch repair
IHC: immunohistochemistry
